# Supplementary material for: Clustering algorithms: A comparative approach
Source: PLoS One. 2019 Jan 15;14(1):e0210236. doi: 10.1371/journal.pone.0210236 (PMC6333366; doi:10.1371/journal.pone.0210236)
Supplement: S1 File — We provide a brief description about the parameters of the clustering algorithms considered in the main text. (PDF) [file pone.0210236.s001.pdf]

Clustering Algorithms: A Comparative Approach

Mayra Z. Rodriguez[1], Cesar H. Comin[2*], Dalcimar Casanova[3], Odemir M. Bruno[4], Diego R. Amancio[1], Luciano da F. Costa[4], Francisco A. Rodrigues[1]

1 Institute of Mathematics and Computer Science, University of São Paulo, São Carlos, São Paulo, Brazil
2 Department of Computer Science, Federal University of São Carlos, São Carlos, São Paulo, Brazil
3 Federal University of Technology, Paraná, Paraná, Brazil
4 São Carlos Institute of Physics, University of São Paulo, São Carlos, São Paulo, Brazil

* Corresponding author
E-mail: chcomin@gmail.com (CHC)

Description of the clustering algorithms' parameters

In the following, we provide a brief description about the parameters of the clustering algorithms considered in the main text. We note that, since some algorithms do not have a default value for the number of clusters, in all cases we set this parameter as the number of clusters in the dataset.

k-means clustering

```
library(stats)
cl <- kmeans(x=dataset, center= k, iter.max = 10,
             nstart = 1, algorithm = 'Hartigan-Wong')$cluster
```

The k-means algorithm used in the main text has the following parameters:

- iter.max: integer, the maximum number of iterations. Default value: 10.
- nstart: integer, indicates how many random sets should be chosen. Default value: 1.
- algorithm: string, implementation of the k-means algorithm to use. Default value: "Hartigan-Wong".
- center: integer, number of clusters. Default value: number of clusters in dataset.

Clustering for large applications (clara)

```
library(cluster)
cl <- clara(x=dataset, k= k, metric = 'euclidean', samples = 5,
    sampsize= min(nrow(dataset), 40 + 2 * k), rngR = FALSE)
    $cluster
```

The algorithm has the following parameters:

- metric: string, specifies the metric to be used for calculating dissimilarities between observations. Default value: "euclidean".

- sample: integer, number of samples to be drawn from the dataset. Default value: 5.
- sampsize: integer, number of observations in each sample. sampsize should be larger than the number of clusters. Default value: $\min(N, 40 + 2k)$, where N is the number of objects.
- rngR: boolean, whether R's random number generator should be used instead of the primitive clara. Default value: false.
- k: integer, the number of clusters. Default value: number of clusters in dataset.

Hierarchical clustering

```
library(cluster)
cl <- cutree(agnes(dataset, metric = 'euclidean', stand = FALSE,
    method = 'average'), k= k);
```

The hierarchical method has the following options:

- metric: string, metric to use for calculating distances between samples. Default value: "Euclidean".
- method: string, clustering method to use. Default value: "average".
- par.method: integer, specifies the parameter for the dissimilarity calculation in some methods. Default value: 0.
- k: integer, the number of clusters. Default value: number of clusters in dataset.

Expectation maximization (EM)

```
library(mclust)
n <- dim(dataset)[1]
init.z <- unmap(sample(1:k, n, replace=T))
mstep <- mstep(modelName="VII", data = dataset, z = init.z)
estep <- estep(modelName="VII", data = dataset, parameters=
    mstep$parameters)
lhood <- data.frame(iter=iter, lhood=estep$loglik)
a = list(estep=estep, mstep=mstep, lhood=lhood[1:iter, "lhood"])
cl <- map(a$estep$z)
```

The algorithm used for expectation maximization clusterization is provided by the *mclust* package. Two routines of the package are used for applying the method:

- mstep: Maximization step in the EM algorithm for parametric Gaussian mixture models.
  - z: string, conditional probability of the i-th observation belonging to the k-th component of the mixture. Default value: "random".
  - modelName: string, indicates the model to be used. Default value: "VII".
- estep: Implements the expectation step of EM algorithm for parameterized Gaussian mixture models.
  - modelName: string, indicates the model to be used. Default value: "VII".
  - parameters: List containing the mean, variance and mixing proportion for each component. These parameters are usually obtained in the expectation mstep.

hcmodel clustering

```
library(mclust);
cl <- hclass(hc(as.matrix(dataset), modelName = 'VVV', use = '
    VARS'), k)
```

Provided by the *mclust* package. The hc routine employing the hcmodel has the following parameters:

- modelName: string, indicates the model to be used. Default value: "VVV".
- use: string, specify what type of data/transformation should be used for model-based hierarchical clustering. Default value: "VARS".
- k: integer, number of clusters. Default value: number of clusters in dataset.

Spectral algorithm

```
library(kernlab);
cl <- specc(as.matrix(dataset), centers = k, kpar = 'automatic',
    kernel = "rbfdot",
nystrom.sample =  dim(dataset)[1]/6, iterations = 200)@.Data
```

The routine specc of the *kernlab* package has the following options:

- centers: integer, number of clusters. Default value: number of clusters in dataset.
- kernel: string, the kernel function used in computing the affinity matrix. Default value: "rbfdot". The following options are available:
  - rbfdot: Radial Basis kernel function ("Gaussian").
  - polydot: Polynomial kernel function.
  - vanilladot: Linear kernel function.
  - tanhdot Hyperbolic tangent kernel function.
  - laplacedot Laplacian kernel function.
  - besseldot: Bessel kernel function.
  - anovadot: ANOVA RBF kernel function.
  - splinedot: Spline kernel.
  - stringdot: String kernel
- kpar: string, the kernel parameter can also be set to a user defined function of class "kernel" by passing the function name as an argument. Default value: "automatic".
- nystrom.sample: float, proportion of data to use when estimating sigma. Default value: $N_b/6$, where N_b is the number of objects.
- iterations: integer, the maximum number of iterations. Default value: 200.

Subspace algorithm

```
cl <- hddc(dataset, K=1:k, model=c(1), algo='EM', init='kmeans',
    mini.nb=c(5,10), min.individuals=2, d_max=100)$class)
```

The routine used for subspace clustering, contained in the *HDclassif* package, is called hddc (High Dimensional Data Clustering). It has the following parameters:

- model: integer or string, 14 models can be used: 12 models with class specific orientation matrix and two models with common covariance matrix. Default value: "akjbkQkdk" or 1.
- k: designates the number of clusters. The algorithm selects the result with the maximum BIC value. Default value: the selected k is in the default interval $(1, 10]$.
- algo: string, the algorithm used for clustering. Can be either EM, CEM (Classification EM) or SEM (Stochastic EM). Default value: "EM".
- init: string, how to the initial class assignments are done. Default value: "kmeans". Four initializations have been implemented:
  - random: each observation is randomly assigned to a class.
  - kmeans: the initial class of each observation is provided by the k-means algorithm.
  - param: initializes according to a multivariate normal distribution.
  - mini-em: the EM algorithm is run m times for nb iterations, the result with the highest likelihood is kept as the initialization of the algorithm.
- mini.nb: integer, used when parameter init is "mini-em". It is an array of length 2 containing m and nb. Default value: (5, 10).
- min.individuals, integer, is used to control for the minimum population of a class. The value of 'min.individuals' cannot be lower than 2

Optics algorithm

```
res_optics <- optics(dataset, eps = 200, minPts= 5)
cl <- extractXi(res_optics, xi = 0.01)$cluster
```

- eps: size of the epsilon neighborhood. This parameter does not have a default value, it was chosen a epsilon neighborhood of 200.
- MinPts: number of minimum points in the eps region (for core points). Default is 5 points.
- Xi: steepness threshold to identify clusters hierarchically based on the steepness of the reachability plot. This parameter does not have a default value, it was chosen 0.01.

Dbscan algorithm

```
cl <- optics(dataset, eps = 0.2, minPts= 5)
```

- eps: size of the epsilon neighborhood. This parameter does not have a default value, it was chosen a value close to the optimal value based in a distance plot.
- MinPts: number of minimum points in the eps region (for core points). Default is 5 points.
