# Supplementary material for: Clustering algorithms: A comparative approach
Source: PLoS One. 2019 Jan 15;14(1):e0210236. doi: 10.1371/journal.pone.0210236 (PMC6333366; doi:10.1371/journal.pone.0210236)
Supplement: S2 File — The file contains figures showing the histograms of ARI values obtained for identifying the clusters of, respectively, datasets DB10C10F and DB2C10F using a random selection of parameters. Each plot corresponds to a clustering method considered in the main text. (PDF) [file pone.0210236.s002.pdf]

Clustering Algorithms: A Comparative Approach

Mayra Z. Rodriguez[1], Cesar H. Comin[2*], Dalcimar Casanova[3], Odemir M. Bruno[4], Diego R. Amancio[1], Luciano da F. Costa[4], Francisco A. Rodrigues[1]

1 Institute of Mathematics and Computer Science, University of São Paulo, São Carlos, São Paulo, Brazil
2 Department of Computer Science, Federal University of São Carlos, São Carlos, São Paulo, Brazil
3 Federal University of Technology, Paraná, Paraná, Brazil
4 São Carlos Institute of Physics, University of São Paulo, São Carlos, São Paulo, Brazil

* Corresponding author
E-mail: chcomin@gmail.com (CHC)

Clustering performance obtained for random selection of parameters

Figures 1, 2, 3 and 4 show the histograms of ARI values obtained for identifying the clusters of, respectively, datasets DB10C10F and DB2C10F using random selection of parameters. Each plot corresponds to a clustering method considered in the main text.

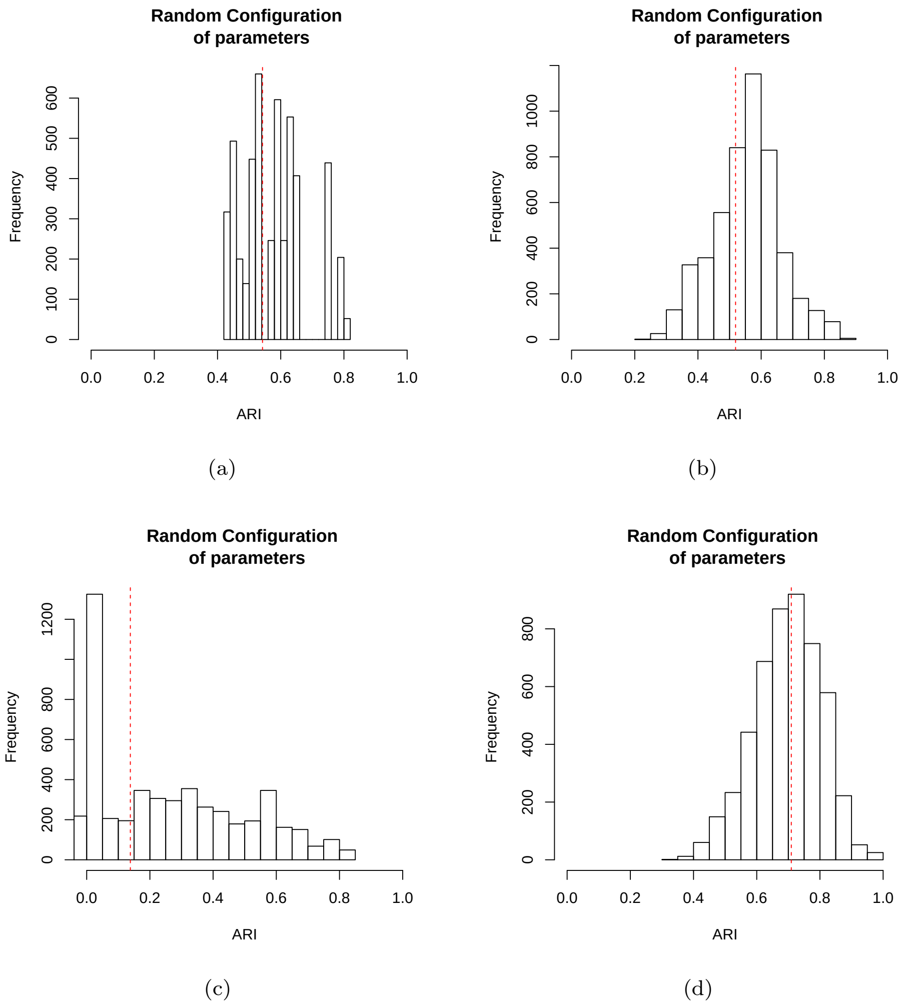

Figure 1. Distribution of ARI values obtained for dataset DB10C10F using random selection of parameters. The distributions correspond to the (a) *hcmodel*, (b) *clara*, (c) *hierarchical* and (d) *spectral* methods. The red dashed line indicates the performance achieved when using the default parameters provided by the respective implementations of the algorithms.

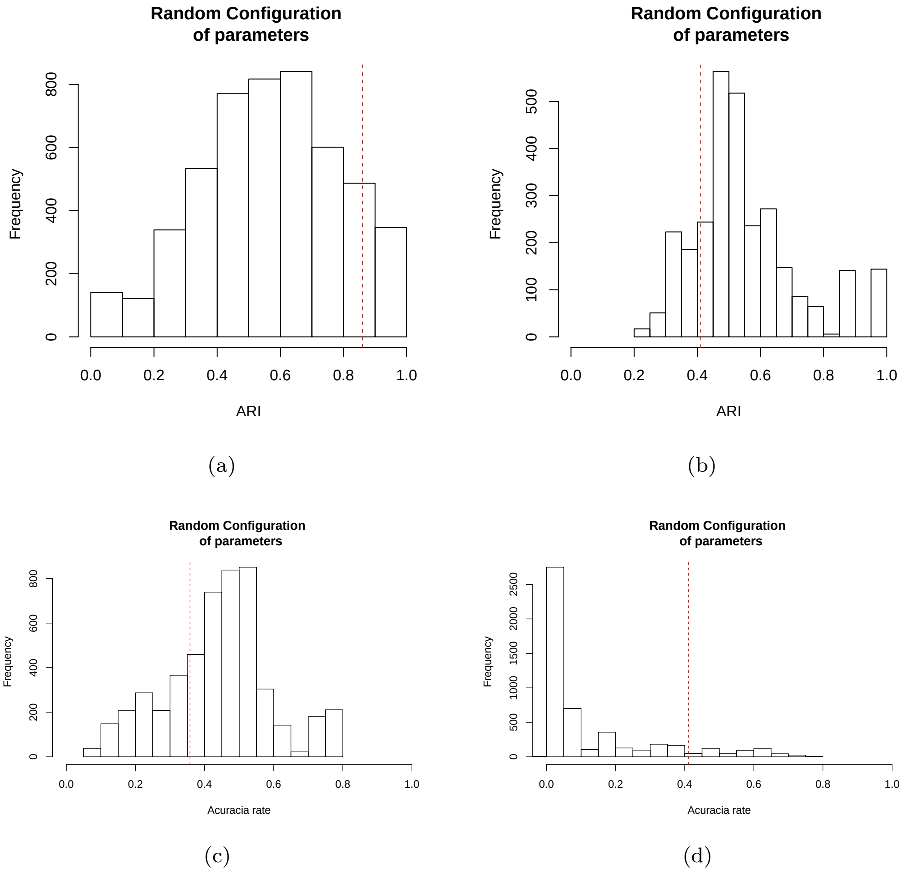

Figure 2. Distribution of ARI values obtained for dataset DB10C10F using random selection of parameters. The distributions correspond to the (a) *Subspace*, (b) *EM*, (c) *optics* and (d) *dbscan* methods. The red dashed line indicates the performance achieved when using the default parameters provided by the respective implementations of the algorithms.

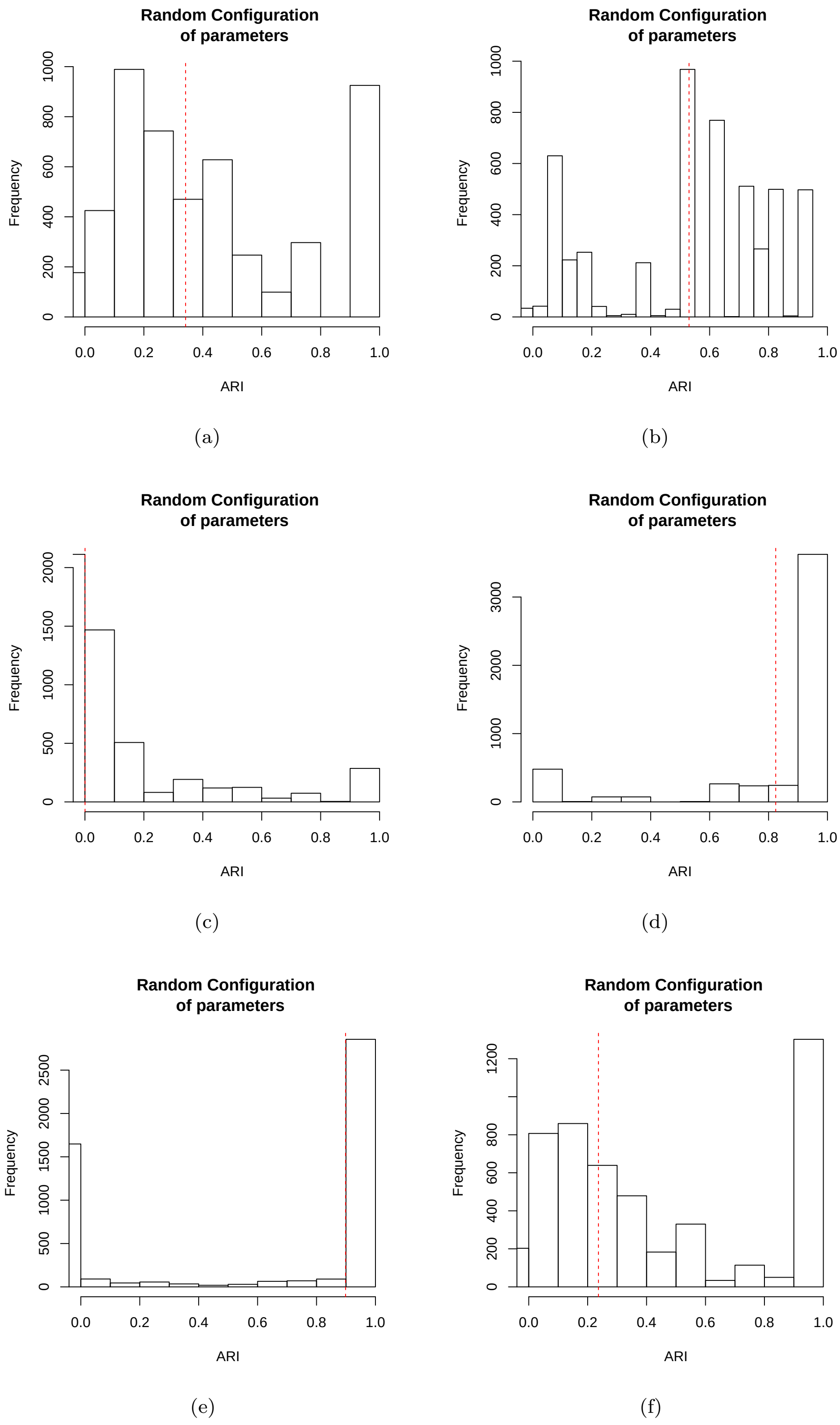

Figure 3. Distribution of ARI values obtained for dataset DB2C10F using random selection of parameters. The distributions correspond to the (a) *hcmodel*, (b) *clara*, (c) *hierarchical* and (d) *spectral* methods. The red dashed line indicates the performance achieved when using the default parameters provided by the respective implementations of the algorithms.

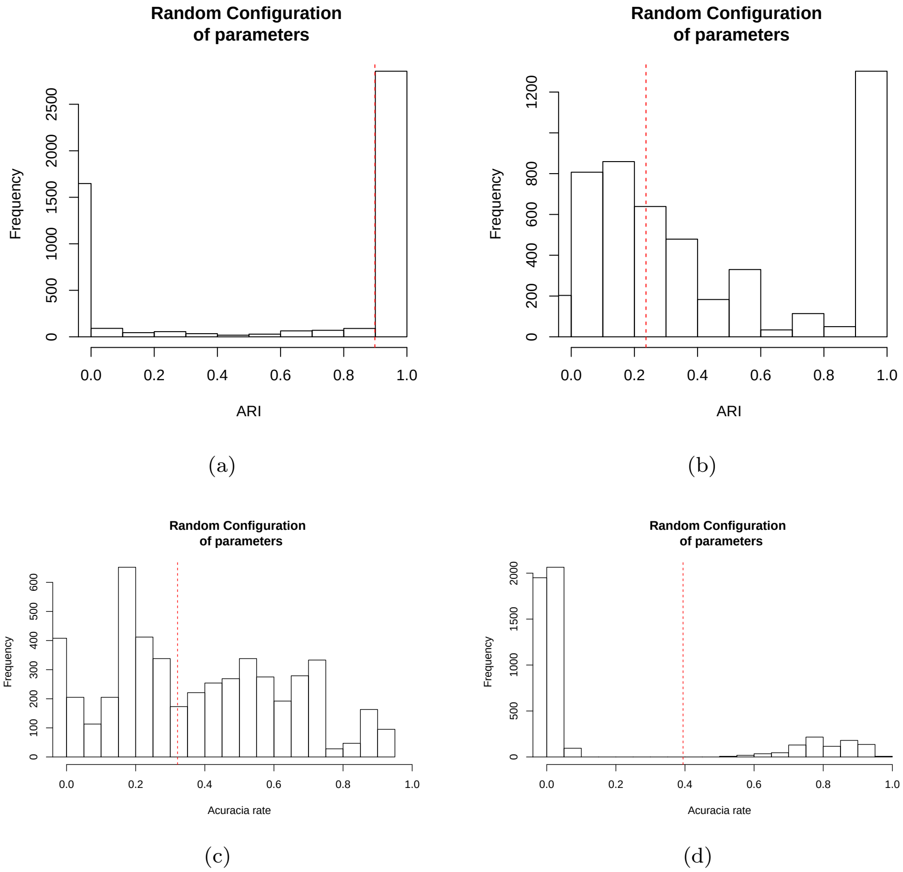

Figure 4. Distribution of ARI values obtained for dataset DB2C10F using random selection of parameters. The distributions correspond to the (a) *Subspace*, (b) *EM*, (c) *optics* and (d) *dbscan* methods. The red dashed line indicates the performance achieved when using the default parameters provided by the respective implementations of the algorithms.
